# The assessment of patients undergoing cardiac surgery for Covid-19: Complications occurring during cardiopulmonary bypass

**DOI:** 10.1177/02676591211018983

**Published:** 2021-05-27

**Authors:** Alfred H Stammers, Linda B Mongero, Eric A Tesdahl, Kirti P Patel, Jeffrey P Jacobs, Michael S Firstenberg, Courtney Petersen, Shannon Barletti, Autumn Gibbs

**Affiliations:** 1 Medical Department, SpecialtyCare, Brentwood, TN, USA; 2 Division of Thoracic and Cardiovascular Surgery, Department of Surgery, University of Florida, Gainesville, FL, USA; 3 Director of Research and Special Projects, William Novick Global Cardiac Alliance, Memphis, TN, USA

**Keywords:** cardiac surgery, cardiopulmonary bypass, COVID-19, complications

## Abstract

The outbreak of the novel coronavirus pandemic (COVID-19) has resulted in dramatic changes to the conduct of surgery both from a patient management perspective and in protecting healthcare providers. The current study reports on the status of COVID-19 infections in patients presenting for cardiac surgery with cardiopulmonary bypass (CPB) on circuit complications. A tracking process for monitoring the presence of COVID-19 in adult cardiac surgery patients was integrated into a case documentation system across United States hospitals where out-sourced perfusion services were provided. Assessment included infection status, testing technique employed, surgery status and CPB complications. Records from 5612 adult patients who underwent cardiac surgery between November 1, 2020 and January 18, 2021 from 176 hospitals were reviewed. A sub-cohort of coronary artery bypass graft patients (3283) was compared using a mixed effect binary logistic regression analysis. 4297 patients had negative test results (76.6%) while 49 (0.9%) tested positive for COVID-19, and unknown or no results were reported in 693 (12.4%) and 573 (10.2%) respectively. Coagulation complications were reported at 0.2% in the negative test results group versus 4.1% in the positive test result group (p < 0.001). Oxygenator gas exchange complications were 0.2% in the negative test results group versus 2.0% in the positive test results group (p = 0.088). Coronary artery bypass graft patients with a positive test had significantly higher risk for any CPB complication (p = 0.003) [OR 10.38, CI 2.18–49.53] then negative test patients [OR 0.01, CI 0.00–0.20]. The present study has shown that patients undergoing cardiac surgery with CPB who test positive for COVID-19 have higher CPB complication rate than those who test negative.

## Introduction

The coronavirus (COVID-19) pandemic has caused unprecedented changes in how healthcare is delivered across the world, with hospitals altering the conduct of services to meet the demands of critically ill patients. Guidelines have been created by regulatory authorities such as the Centers for Disease Control and Prevention and the European Center for Disease Prevention and Control that provide recommendations for healthcare facilities and workers.^1,2^ Non-urgent surgeries have been delayed to conserve resources and assuage the stresses experienced by an overburdened hospital staff. Despite the efforts of hospitals and healthcare providers some patients may delay their care related to concerns of entering a health system in the middle of a pandemic. While professional societies have recommended triaging cardiac surgical patients^3,4^ it is often difficult to assess how to quantify the urgency of the cardiac disease process in which delays may result in cardiac decompensation.

Population-based diagnostic testing is a critical component in understanding the spread of COVID-19 and in establishing mitigation processes. While this has been fraught with difficulties that include access to test facilities, inaccurate results and asymptomatic COVID-19 infected individuals, it remains the most effective means in reducing the spread of the virus.^5^ The Society of Thoracic Surgeons COVID-19 taskforce has recommended that testing for the virus be completed within 48 hours of surgery and then again on admission.^6^ While those patients who test negative for COVID-19 within this time frame may proceed with surgery, those testing positive should have their surgery delayed if at all possible.^7,8^ It has been shown that outcomes of cardiac surgical patients who contract COVID-19 in the perioperative period are extremely poor with extended lengths-of-stay and high mortality.^9^ It is conceivable that the pathophysiologic responses seen in infected patients may make the conduct of cardiopulmonary bypass (CPB) more difficult.^10^ The present study reports the results of a national registry on testing for COVID-19 and complications that arise during extracorporeal flow in patients undergoing cardiac surgery with CPB.

## Methods

The study was conducted by reviewing records from patients who underwent cardiac surgery with the use of CPB and were in the SpecialtyCare Operative Procedure rEgistry (SCOPE™) which has been previously described (SpecialtyCare is a United States provider of Allied Health services, and the SCOPE™ Registry contains data from over 1 million perfusion procedures in over 40 states at more than 300 hospitals [https://specialtycareus.com/]).^11,12^ The SCOPE™ registry was established as a national quality control database for systematically collecting intraoperative data from cardiac surgical procedures, and serves a multifunctional purpose focused on performance improvement. Its goal is to achieve the following: standardization of electronic data recording of specific perioperative quality indicators; creation of reporting tools including dashboards and written reports; benchmarking of performance at multiple levels including the clinician, the hospital, and geographical region. Over 100 quality indicators are utilized for data analytics and each indicator is regularly reviewed by an advisory board who use the best available evidence to assess which data points should be acquired. The system uses a proprietary software application (Case Documentation System, SpecialtyCare, Brentwood, TN, USA ) that records demographic and intraoperative data for every case, and consists of nearly 2 million cases with one quarter being cardiac surgery procedures. Data validation is assured by monthly auditing of random case records (a minimum of three records for each perfusionist per quarter) with the results analyzed and reported by individual clinicians. Deviations are reviewed and corrections made to the central database. The data is updated daily and is presented by the use of dashboards with the analytic assessment at multiple levels of performance. Institutional ethics review board approval (Protocol # 12017, ADVARRA, Center for IRB Intelligence, 6940 Columbia Gateway Drive, Suite 110, Columbia, MD, 21046, USA) was obtained for this study.

Cases conducted between November 1, 2020 through January 18, 2021 at 176 hospitals throughout the United States and Puerto Rico were reviewed. During the study period all surgical patients over 18 years of age who underwent a cardiac procedure requiring CPB were included. Patients were excluded from the analysis if they did not have all required quality indicators recorded or were missing data. Groups were established based upon the assessment and result of COVID-19 testing. The four groups were negative test (Neg Test), positive test (Pos Test), unknown test (Unk Test) and no test (No Test). The primary end point was any complication during CPB with secondary end points of coagulation and gas exchange complications.

Soon after the onset of the COVID-19 pandemic a modification was made for documenting case information. A series of eight new questions were added that were related to the COVID-19 status (Appendix 1). These questions were focused on COVID-19 assessment and the status of the surgery if a positive test was confirmed. Perfusionists were also asked three questions on complications specifically on coagulation, oxygenator gas exchange performance or any complication during CPB. Each question contained a definition which was constantly displayed so that the clinician could view what was being asked. A drop-down menu was used for selection of a response for each question. To improve clarity the clinician was given the names of individuals within the medical department to contact for further delineation. The process for collecting this data were started in May 2020 and assessed and revised through October 2020. Therefore, the start of data collection for analysis began on November 1, 2020.

**Figure 1.**
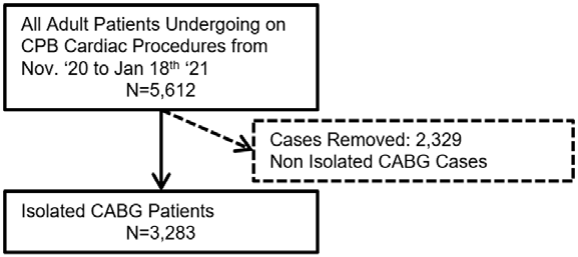
Flowchart for patient selection for regression analysis. CABG: coronary artery bypass graft; CPB: cardiopulmonary bypass.

To improve the homogeneity of the study population regression analyses were performed on first-time coronary artery bypass graft (CABG) patients. These patients were used for the regression model and controlled for factors described below. The flowchart for patient selection for the regression model is shown in Figure 1.

### Statistical analysis

Descriptive statistics were calculated as count and percentage for categorical variables, mean and standard deviation for continuous variables. Data were described as mean and standard deviation unless otherwise specified. Unadjusted group differences were assessed using chi-squared tests, and Welch’s analysis of variance.

Two rudimentary mixed effects regression analyses were estimated to assess the likelihood of any complication and average heparin dose, respectively. These models were estimated on a subset of isolated CABG patients to assess the possible effects of COVID-19 test results while controlling for age, gender and body mass index, as well as a random effect controlling for surgeon. Variables with missing data were excluded from all analyses. All analyses were completed using the R statistical computing environment^13^ in conjunction with the “tableone,” “lme4,” “sjPlot” packages.^14–16^

## Results

**Figure 2.**
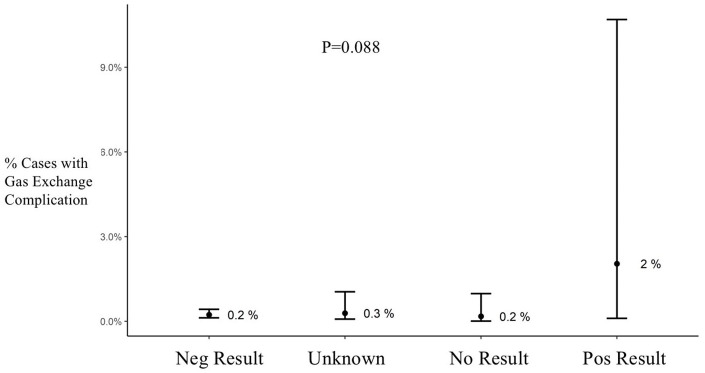
Oxygenator gas exchange complications during cardiopulmonary bypass by test group. Results are shown as average and confidence interval. Neg: negative; Pos: positive.

A total of 5612 records were reviewed with the distribution of cases by test result shown in Figure 2. Of the patients tested the majority were assessed by nasopharyngeal swab (94.0%) while antigen testing was infrequently reported (6.0%). The majority of patients had been tested for COVID-19 prior to surgery (77.5%) with the Neg Test group having the highest percent (76.6%) of tested patients. A large number of patients were in the Unk and No Test groups (22.6%). A total of 49 patients (0.9%) had a positive test for COVID-19. Of these 31 (63.3%) did not have their surgery delayed, while 15 (30.6%) had the surgery delayed for 14 days, and three (6.1%) were delayed until a negative test was obtained. Patients were classified by surgery class using the Society of Thoracic Surgeons definitions as elective, emergent salvage, emergent or urgent (Protocol # 12017, ADVARRA, Center for IRB Intelligence, 6940 Columbia Gateway Drive, Suite 110, Columbia, MD, 21046, USA). For the Pos Test group surgery was delayed for elective procedures18.4% (9) of the time, emergent surgery 2.0% (1) and urgent surgery 16.3% (8). For patients in either the Unk Test or No Test groups 77.1% (965) were classified as elective, 9.4% (118) as emergent, 1.0% (13) as emergent salvage and 13.6% (170) as urgent.

The primary surgical procedure performed was a CABG which ranged from 58.3% in the Unk Test group to 71.4% in the Pos Test group (Table 1). While there was no intergroup difference in patient age, there were significantly more women in the Pos Test group when compared to the others with similar findings for weight (Table 2). The Pos Test group had the lowest on-CPB hematocrit and the highest red blood cell transfusion rate.

**Table 1.** Distribution of cases by procedure type.

Procedure type	All (%)	Neg test (%)	Unk test (%)	No test (%)	Pos test (%)
	*N* = 5612	*N* = 4297	*N* = 693	*N* = 573	*N* = 49
Aortic surgery	272 (4.85)	183 (4.26)	56 (8.08)	29 (5.06)	4 (8.16)
AV surgery + CABG	309 (5.51)	239 (5.56)	40 (5.77)	30 (5.24)	0 (0.00)
CABG reoperation	42 (0.75)	34 (0.79)	5 (0.72)	2 (0.35)	1 (2.04)
AV/MV surgery	83 (1.48)	62 (1.44)	14 (2.02)	7 (1.22)	0 (0.00)
Isolated AV surgery	395 (7.04)	302 (7.03)	47 (6.78)	43 (7.50)	3 (6.12)
Isolated CABG	3536 (63.0)	2726 (63.5)	404 (58.3)	371 (64.7)	35 (71.4)
Isolated MV surgery	360 (6.42)	288 (6.70)	39 (5.63)	31 (5.41)	2 (4.08)
MV surgery + CABG	102 (1.82)	78 (1.82)	18 (2.60)	5 (0.87)	1 (2.04)
Other	502 (8.95)	375 (8.73)	69 (9.96)	55 (9.60)	3 (6.12)
Unknown	10 (0.18)	9 (0.21)	1 (0.14)	0 (0.00)	0 (0.00)

Data are presented as number and percent.

AV: aortic valve; CABG: coronary artery bypass graft; MV: mitral valve; Neg: negative; Pos: positive; Unk: unknown.

**Table 2.** Demographic and intraoperative results by study group.

	All	Neg test	Unk test	No test	Pos test	*p* Value
	*N* = 5612	*N* = 4297	*N* = 693	*N* = 573	*N* = 49	
Gender, *N* (%)						0.006
Men	4058 (72.3)	3149 (73.3)	483 (69.7)	398 (69.5)	28 (57.1)	
Women	1554 (27.7)	1148 (26.7)	210 (30.3)	175 (30.5)	21 (42.9)	
Patient age (years), mean (SD)	64.3 (12.5)	64.2 (12.6)	64.3 (12.2)	65.2 (12.2)	65.3 (10.6)	0.34
Patient weight (kg), mean (SD)	88.3 (20.8)	88.7 (20.9)	88.2 (20.5)	86.3 (20.5)	84.7 (15.0)	0.041
BMI (kg/m^2^), mean (SD)	29.9 (6.9)	30.0 (6.7)	30.0 (8.9)	29.2 (5.9)	29.5 (5.6)	0.084
Lowest CPB Hct, mean (SD)	25.9 (4.8)	26.0 (4.8)	25.7 (4.6)	25.3 (4.8)	24.1 (4.6)	<0.001
Intraop RBC transfusion, *N* (%)						0.019
No	3986 (71.2)	3060 (71.4)	481 (69.7)	419 (73.3)	26 (53.1)	
Yes	1609 (28.8)	1224 (28.6)	209 (30.3)	153 (26.7)	23 (46.9)	
Total heparin during CPB (KIU), mean (SD)	17.6 (13.6)	18.3 (14.1)	16.1 (13.2)	14.3 (9.8)	14.0 (9.9)	<0.001
Total heparin during CPB (KIU), median [25th, 75th percentile]	13.0 [10.0;20.0]	15.0 [10.0;25.0]	10.0 [10.0;20.0]	10.0 [10.0;20.0]	10.0 [10.0;20.0]	<0.001
ACT during CPB (seconds), *N* (%)						0.419
<400 seconds	66 (1.18)	49 (1.14)	11 (1.59)	5 (0.87)	1 (2.04)	
>400 seconds	5546 (98.8)	4248 (98.9)	682 (98.4)	568 (99.1)	48 (98.0)	

ACT: activated clotting time; BMI: body mass index; CPB: cardiopulmonary bypass; Hct: hematocrit; KIU: thousands per international unit; Neg: negative; Pos: positive; RBC: red blood cell; SD: standard deviation; Unk: unknown.

**Figure 3.**
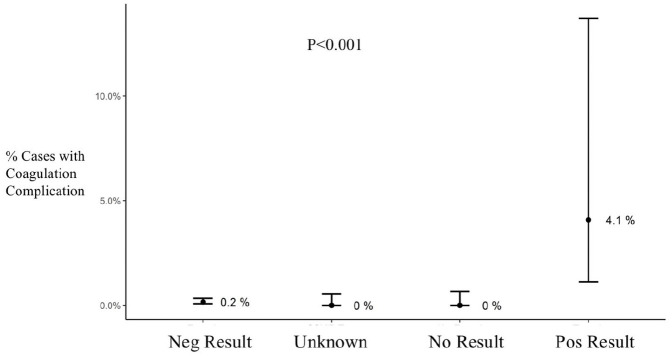
Coagulation complications during cardiopulmonary bypass by test group. Results are shown as average and confidence interval. Neg: negative; Pos: positive.

Bivariate analysis showed that while there was a trend towards higher oxygenator gas exchange complications in the Pos Test group, this finding was not statistically reliable (p = 0.088) (Figure 2). There was a higher incidence of coagulation complications in the Pos Test group compared to all other groups (p < 0.001) (Figure 3). This group also had an average of 4300 IU less heparin administered on CPB than the Neg Test group (p < 0.001) (Table 2). There were no differences in the number of patients that had activated clotting time (ACT) values less than 400 seconds during CPB.

**Figure 4.**
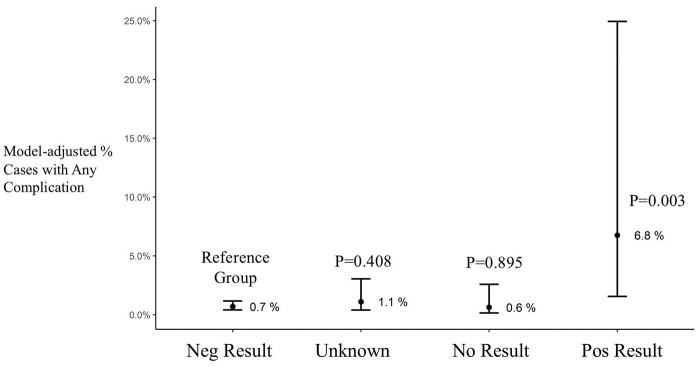
Model adjusted cases with any complication during cardiopulmonary bypass. Results are shown as average and confidence interval. Neg: negative; Pos: positive.

**Figure 5.**
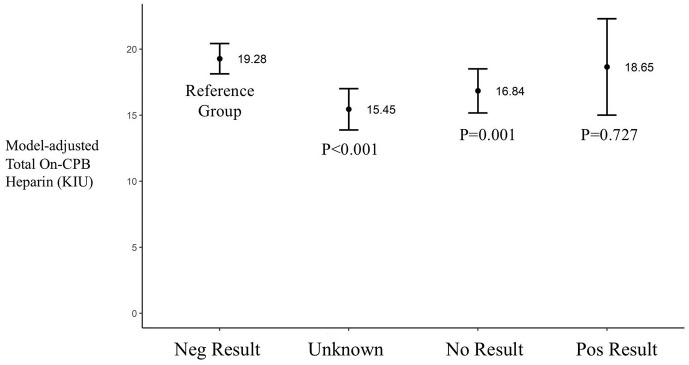
Model adjusted total on-CPB heparin between groups. Results are shown as average and confidence interval. Neg: negative; Pos: positive.

There were 3283 patients in the CABG only analysis distributed as follows: Neg Test group 2359 patients, Unk Test group 376 patients, No Test group 337 patients and Pos Test group 31 patients. The results of regression model for CABG patients are shown in Table 3. Having a positive COVID-19 test considerably increased the odds of any complication during CPB (OR, 10.38; 95% CI, 2.18–49.53; p = 0.003). When controlling for multiple variables the results of any complication during CPB occurred 0.7% in the Neg Test group and 6.8% for the Pos Test group (p = 0.003, Figure 4). The average total heparin given on CPB was not different between Neg Test and Pos Test groups (p = 0.727, Figure 5).

**Table 3.** Regression-adjusted odds of developing any complication during cardiopulmonary bypass relative to negative COVID-19 test result.

Predictors	Any new complication		
	Odds ratio	CI	*p* Value
COVID-19 Neg test	0.01	0.00–0.20	0.004
COVID-19 Unknown test	1.6	0.52–4.89	0.408
COVID-19 No test	0.9	0.20–4.03	0.895
COVID-19 Pos test	10.38	2.18–49.53	0.003

Neg: negative; Pos: positive.

## Discussion

Preoperative testing for COVID-19 for cardiac surgery is strongly recommended to detect patients who may be asymptomatic as a means to protect both patients and healthcare providers.^17^ When a positive test is confirmed the decision to proceed with surgery must be carefully weighed against the risk for complications related to the infection. Individuals with cardiovascular disease who test positive are especially at risk for poor outcomes as a result of their underlying disease and by the virulence of COVID-19.^18^ Adding the cumulative deleterious effects of surgery and CPB exacerbates the injury further.^19^ While it is unknown on how long to postpone cardiac surgery in asymptomatic patients deemed non-emergent, some advocate for a minimum wait period of two to four weeks after a positive COVID-19 test and the absence of symptoms.^8^ In our study the majority of patients who tested positive for COVID-19 (63.0%) did not have their surgery delayed. The outcomes of cardiac surgical patients who were diagnosed with COVID-19 infection in the early postoperative period has been shown to be extremely poor.^9^ In this study nine of 97 cardiac surgical patients tested positive for COVID-19 with four (44%) dying before leaving the hospital, as opposed to 5.5% of patients operated concurrently who were not infected. The authors explained that the hospital did not have an established screening protocol for COVID-19 at the time of surgery, so they speculated that the patients became infected either during the preoperative period or while in the hospital. They further stated that the clinical sequelae only appeared in the postoperative period.

While there has yet to be a large study published on the effects of COVID-19 and cardiac surgery, an international multicenter study on surgical patients who tested positive for COVID-19, either seven days before surgery or 30 days thereafter, revealed significantly higher pulmonary complications and mortality, which was especially prevalent in men 70 years and older.^20^ In a small study of 25 asymptomatic patients with COVID-19 who underwent cardiac surgery there was a higher rate of postoperative respiratory dysfunction and death in patients who required readmission to the intensive care unit when compared to those who did not.^21^ In that same study the authors used a non-COVID-19 propensity-matched group to positive tested patients and found higher lengths-of-stay and mortality in infected patients.

While large cardiac surgery case series are lacking, a number of case reports have appeared. Fukuhara reported on an urgent patient who presented with an aortic dissection who required immediate surgery.^22^ The patient was not tested in the preoperative period because he did not meet the criteria in use at that time at the hospital. The operative course was uneventful but during the recovery period that patient had worsening respiratory function, and on the fifth postoperative day (POD) a reverse transcriptase polymerase chain reaction (RT-PCR) test for COVID-19 was performed and was positive. The patient died on the POD 11 of multisystem organ failure. While several reports emphasize the susceptibility of COVID-19 patients to postoperative complications, there have been reports of successful intervention. Mori et al.^23^ reported successful outcomes in two patients requiring urgent surgery for aortic dissections with one testing positive prior to surgery and the other two months post-discharge. In a similar report Martens et al.^24^ described the course of a patient with an aortic dissection who tested positive in the postoperative period and had an unremarkable recovery untill the POD 6 when mild respiratory symptoms appeared. However full resolution occurred and the patient was discharged on the 14th day without supplemental oxygen.

In the present study we reviewed complications that included disturbances to coagulation and/or gas exchange during extracorporeal support in patients who have undergone cardiac surgery with CPB during the COVID-19 pandemic. While the incident rate of complications is low there appears to be a trend towards increased circuit problems in patients who have tested positive for the virus. While the reasons for this are unknown they may be related to the pathogenesis of the coronavirus disease process complicated by the intricacies of cardiac surgery and the inflammatory response system induced by CPB. It has been shown that coronavirus infection causes an enhanced inflammatory response with hypercytokinemia,^25^ as well as hematological disturbances that present as abnormalities to the coagulation system.^26,27^ The latter is especially notable since cardiac surgery patients may have prothrombotic underlying conditions related to cardiovascular disease. The use of anticoagulant and platelet inhibiting medications are frequently seen in this patient population which further impairs the hemostatic response. COVID-19 induces a hypercoagulable state which places patients at an increased thrombophilic risk and has been shown to result in both venous and arterial thrombosis.^28^ In light of this it is not unreasonable to expect a hypercoagulable response to also occur during CPB. It has been suggested that because of this heightened coagulation response to assure that all extracorporeal surfaces in contact with blood are modified through surface treatment to reduce thrombogenicity, and to carefully monitor anticoagulation.^10^ It was for these reasons that we chose to modify our data collection system to quantify complications such as oxygenator gas exchange and coagulation disturbances that may occur during CPB in the era of COVID-19.

In the present study a gas exchange complication was defined as one that resulted in higher than expected FiO2 level to maintain oxygenation, or a higher than normal gas flow sweep rates to maintain normocarbia, or a combination of the two. Coagulation disturbances were defined as visual observation of clots in the field or circuit during systemic heparinization, heparin resistance with the requirement of high heparin utilization, or a combination of the two. We also chose to include a category of ‘any complication’ to quantify a CPB incident that was not specific to either gas exchange or coagulation disturbance. While this definition is broad we included it to allow for the recording of events that were related to CPB but not constrained by a specific definition. One patient who tested positive for the virus and did not have a delay in surgery experienced both gas exchange and coagulation complications, while a second patient who had surgery delayed for 14 days was found to be heparin resistant. No further assessment of the heparin resistance was made (antithrombin III level, heparin induced thrombocytopenia) so it is impossible to discern the exact cause. The total heparin administered during CPB was significantly lower in the Pos Test Group as compared to those patients with a negative test result (p = 0.001), but there were no differences in the number of patients with on-CPB ACT values under 400 seconds. There were significantly more women in the Pos Test group then Neg Test with a concomitant lower weight by an average of 4 kg (p = 0.041). While it is unknown why there was higher quantities of heparin given during CPB, it may be related to a gender related difference in pharmacokinetic activity of heparin.^29^ When controlling for multiple variables the model-adjusted average total heparin given on CPB was not statistically different between the Neg Test and Pos Test groups (p = 0.727).

Complications during CPB are rare events which is testament to the continuous improvement in techniques and technologies that influence the conduct and safety of perfusion.^30^ While the overall percent of COVID-19 patients with positive tests was low (0.9%), the complication rate was ten-fold higher than all other groups. A number of studies have shown that COVID-19 infected patients experience higher complication rates and mortality, but none have reported on intraoperative incidents or those that occurred during CPB.^9,18,19^ Our study only reviewed intraoperative events and did not follow these patients postoperatively. In a study that we conducted on COVID-19 patients supported by extracorporeal membrane oxygenation (ECMO) we found that more than 30% of all patients experienced circuit complications that necessitated replacing the entire circuit or integral components.^31^ The most often reported reason for these change-outs were thrombosed circuits as well as problems with oxygenation and/or carbon dioxide removal. While a comparison between ECMO patients and those undergoing cardiac surgery is inappropriate, the fact that a similar trend is seen with both applications of extracorporeal circulation warrants further examination of the influence of COVID-19 on complications.

In order to further study the risk of complications we chose to perform a sub-analysis on patients undergoing isolated CABG surgery. This was done to reduce the heterogeneity associated with multiple surgical procedures. By doing so, and controlling for multiple factors, we found that the Pos Test group was more than ten times more likely to have any complication during CPB then the Neg Test group. While a comparison of all patients has shown significantly more heparin given on CPB in the Neg Test group, this was not seen by multivariable regression analysis where no difference was observed between the Neg Test and Pos Test groups (p = 0.727).

Also of concern is the large number of patients with either an unknown or absent test result is concerning. While the incident rate was higher in these groups then the Neg Test patients, without knowledge of their test status no inference can be made. The fact that the healthcare providers did not know the status of nearly a quarter of patients undergoing surgery is worrisome.

### Study limitations

There are several limitations to this study. The low occurrence rate of circuit complications may influence the outcome since only two patients in the Pos Test group showed complications. We are continuing to collect data and will assess if these trends continue especially in light of a growing population of patients who are being infected with COVID-19. A second issue concerns how individual perfusionists who loaded the case information interpreted the definitions for complications. We began collecting data in May 2020 and made changes to the way the queries were stated in the case documentation system and improved the definitions before the start of this study (November, 2020). We cannot rule out that some associates may have misinterpreted the questions. A third issue includes the analysis of data from a retrospective observational data registry. While data were collected in a prospective manner it is nonetheless non-randomized. Registry data does not permit the investigation of certain data that may be pertinent in determining further effects not found with limited variable analysis. Differences in practice patterns do exist and although we attempted to minimize these by multivariable logistic regression, we realize that variability still is present. Furthermore, transfusion guidelines were not standardized across and within individual hospitals so the administration of RBC may have been biased by clinical decisions. The retrospective study design is subject to limitations of inherent selection bias, and the reported results are limited to describe observed associations between the implementation of the described protocol and the improved patient outcomes and do not demonstrate a direct cause-and-effect relationship. However, the reported results were adjusted for the confounding influences. This was not a longitudinal study so the effect of complication or patient outcomes in the postoperative period were not measured. And finally, there exists a potential for the miscoding of data, which despite steps for validation, must be considered in any secondary analysis of a registry data.

## Conclusions

While the present study raises important questions regarding and increased risk for complications developing during cardiopulmonary bypass in COVID-19 patients, further studies are required to gain a more cohesive understanding of the effect of this disease on the conduct of extracorporeal circulation. At a minimum those individuals who are involved in the application of extracorporeal circulation would benefit by prophylactically reviewing techniques for managing complications that may arise in COVID-19 infected patients.

## Supplemental Material

sj-pdf-1-prf-10.1177_02676591211018983 – Supplemental material for The assessment of patients undergoing cardiac surgery for Covid-19: Complications occuring during cardiopulmonary bypass Click here for additional data file. Supplemental material, sj-pdf-1-prf-10.1177_02676591211018983 for The assessment of patients undergoing cardiac surgery for Covid-19: Complications occuring during cardiopulmonary bypass by Alfred H Stammers, Linda B Mongero, Eric A Tesdahl, Kirti P Patel, Jeffrey P Jacobs, Michael S Firstenberg, Courtney Petersen, Shannon Barletti and Autumn Gibbs in Perfusion
