# Supplementary material for: The assessment of patients undergoing cardiac surgery for Covid-19: Complications occurring during cardiopulmonary bypass
Source: Perfusion. 2021 May 27;37(4):350–8. doi: 10.1177/02676591211018983 (PMC9069560; doi:10.1177/02676591211018983)
Supplement: sj-pdf-1-prf-10.1177_02676591211018983 – Supplemental material for The assessment of patients undergoing cardiac surgery for Covid-19: Complications occuring during cardiopulmonary bypass [file sj-pdf-1-prf-10.1177_02676591211018983.pdf]

Appendix.

Appendix.

Figure 1. Query list with definitions for COVID-19 in case documentation system.

COVID-19 Assessment	Did the patient have preoperative testing for COVID-19?<br>If so, what was the result?	No<br>Unknown<br>**Yes – Negative Result**<br>**Yes – Positive Result**
COVID-19 Test Type	If the patient was tested for COVID-19, what type of test was administered?	Antigen<br>N/A<br>Nasal Swab<br>Other
COVID-19 Surgery Status	If the patient tested positive for COVID-19 initially, was the surgery delayed?	N/A<br>No<br>Yes – For 14 days<br>Yes – Until tested negative
COVID-19 Surgery Post Initial Delay	If the surgery delayed, was the surgery performed after the patient tested negative for COVID-19?	N/A<br>No<br>Unknown<br>Yes
Complications	Were there any complications encountered in the intraoperative period?	No<br>Yes
Complication: Coagulation	Were there any complications encountered in the intraoperative period?	No<br>Yes – Clots in circuit of field<br>Yes – Heparin resistant<br>Yes – Both HR and clots circ. or field
Complication: Gas Exchange	Was there a complication with gas exchange during CPB?	No<br>Yes – Higher than expected FiO2 norm PaCO2<br>Yes – Higher than expected sweep norm PaCO2<br>Yes – Both high FiO2 and sweep
